# Inferring Functional Brain States Using Temporal Evolution of Regularized Classifiers

**DOI:** 10.1155/2007/52609

**Published:** 2007-09-03

**Authors:** Andrey Zhdanov, Talma Hendler, Leslie Ungerleider, Nathan Intrator

**Affiliations:** ^1^Functional Brain Imaging Unit, Tel Aviv Sourasky Medical Center, 6 Weizmann Street, Tel Aviv 64239, Israel; ^2^The School of Computer Science, Tel Aviv University, P.O. Box 39040, Tel Aviv 69978, Israel; ^3^Psychology Department and Sackler Faculty of Medicine, Tel Aviv University, Tel Aviv 69978, Israel; ^4^Laboratory of Brain and Cognition, National Institute of Mental Health (NIMH), National Institute of Health (NIH), Bethesda, MD 20892-1366, USA

## Abstract

We present a framework for inferring functional brain state from electrophysiological (MEG or EEG) brain signals. Our approach is adapted to the needs of functional brain imaging rather than EEG-based brain-computer interface (BCI). This choice leads to a different set of requirements, in particular to the demand for more robust inference methods and more sophisticated model validation techniques. We approach the problem from a machine learning perspective, by constructing a classifier from a set of labeled signal examples. We propose a framework that focuses on temporal evolution of regularized classifiers, with cross-validation for optimal regularization parameter at each time frame. We demonstrate the inference obtained by this method on MEG data recorded from 10 subjects in a simple visual classification experiment, and provide comparison to the classical nonregularized approach.

## 1. INTRODUCTION

Historically, the goal of inferring person's functional state from brain signals on a single-trial basis was most extensively pursued in the field of EEG-based brain-computer interface (BCI) design [1, 2]. EEG-based BCI systems attempt to distinguish among a small number of consciously controllable mental states from accompanying EEG signals, using the response potential evoked by the stimulus [3, 4]. This approach is often based on machine learning principle using a set of labeled examples to construct a (usually linear) classifier. First BCI experiments utilized a single-trial ERP setup in which subject was presented with stimuli in a controlled fashion and communicated his or her decision by changing mental state (e.g., focus of attention) [3]. Another approach to BCI design attempts to infer subject's mental state exclusively from EEG signals without relying on pacing cues [5–7]. Typically, this free-paced BCIs would split ongoing EEG activity into short (usually less than 1 second) intervals and examine each interval independently in search of EEG patterns, characteristic of one of the predefined mental states.

A wide variety of different algorithms utilizing different features of EEG signal were proposed over the last three decades. The simplest ones like the one described in [8] rely on subjects learning to control their cortical potentials at certain electrode locations, thus reducing the classification algorithm to simple thresholding. More complex algorithms use spatial [9] or spatio-temporal [5–7, 10, 11] features of the EEG signal in conjunction with some classification techniques. Typically, these algorithms treat either raw EEG data or energy of some predefined frequency bands (such as motor-related *μ* and β rhythms) as features. Those features are then fed into some classifiers to produce the final classification. Most BCIs use a variation of a linear classifier such as regularized fisher linear discriminant analysis (LDA) [5], common spatial patterns [9], or support vector machines (SVM) [12]. Some attempts are also made to address the problem with nonlinear classifiers such as artificial neural networks [11]. An extended discussion on comparative merits of linear and non-linear methods can be found in [13].

One type of EEG signal features particularly widely used in BCI is the amount of energy in a certain frequency band. Large neuronal populations are capable of generating large-scale synchronized oscillatory electrical activity observable by EEG. As a general rule, the frequency of such oscillatory activity is believed to decrease as the number of neuronal assemblies forming the network increases [14]. This activity is transient and can be evoked (event-related synchronization, ERS) or suppressed (event-related desynchronization, ERD) by various experimental events such as stimulus presentation. Two particular frequency bands —the Rolandic μ rhythm (7–13Hz) and the central β rhythm (above 13Hz) —are particularly useful for BCI design as they are amenable to conscious control by means of motor imagery (see [15, 16]). More extensive discussion of the ERS/ERD phenomenon can be found in [4].

Current BCI systems are capable of achieving typical classification accuracies in the range of 80–95% for a two-outcome classification trial (one exception is a report in [17] of 100% classification accuracy over 160 trials).

Recently, application of mental state inference techniques to brain research received a lot of attention from the fMRI community [18–21]. While it has been a valuable tool in investigation of endogenously triggered changes of mental states such as bistable perceptual phenomena, it suffers from low temporal resolution. Unlike fMRI, electrophysiological measurements (EEG and MEG) provide a rich source of temporal information; therefore, it is expected that the analysis of the temporal evolution of these signals can be used for fine temporal mental state inference. While mental state inference from EEG signals has been researched extensively in the BCI context, there is little investigation into EEG- and MEG-based inference as a functional neuroimaging research technique.

To be useful outside the BCI domain, inference techniques need to satisfy a set of requirements that differs significantly from the requirements of the BCI design.

1. The choice of functional states that need to be distinguished is often outside the experimenter's control.
2. The subject is not trained to improve the inference accuracy.
3. The inference techniques need to be applicable to modalities other than EEG. In particular, inferring functional states from MEG or fMRI signals raises two major problems: (a) the dimension of input data is much higher than that of EEG and (b) due to technical and cost limitations, the amount of available data is much smaller.
4. The inference method attempts to provide a physiologically meaningful interpretation of the inference criteria.
5. Unlike with BCI, the experimenter has greater control over the experimental environment, making scenarios that require relatively complicated setups (for example, single-trial evoked response potentials (ERPs) experiments) much more attractive.

These differences require a more high-dimensional and robust classifiers than those used for BCI. In addition, the scarcity of data for MEG and fMRI modalities means that more advanced model validation techniques (such as cross-validation, bootstrapping, etc.) are needed.

In this work, we describe a framework for inference of the temporal evolution of functional states. We formulate the inference problem as that of discriminating between two classes of signals time locked to experimental events. Central concepts of the proposed framework are the temporal evolution of regularized linear classifiers constructed from instantaneous signal values and their relation to the regularization parameter. We investigate the behavior of these quantities on MEG dataset from a simple classification experiment that involves switches between two stimulus categories. We construct a classifier by choosing the combination of timepoint and regularization parameter that jointly minimize estimated misclassification rate and analyze the classifier's performance.

## 2. MEG EXPERIMENTAL SETUP

**Figure 1:**
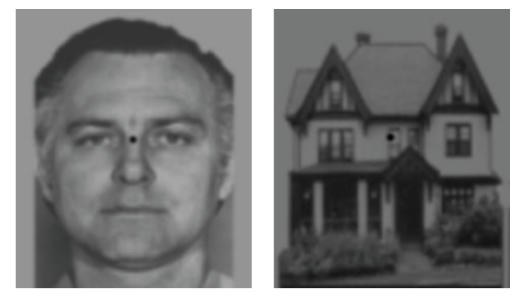
Examples of the stimulus category presented to the subjects.

The MEG experiment was performed on 10 healthy volunteer subjects at the Lab of Brain and Cognition, National Institute for Mental Health (NIMH), Bethesda, Maryland. The study was approved by the Institutional Review Board committee of the NIMH. During the experiment, MEG signals were recorded while subjects were presented with images from two different categories —faces and houses. The images of faces were taken from the Ekman and Friesen [22] and KDEF [23] databases and were composed of 4–6 female or male particulars exhibiting fearful or neutral facial expression (for an example of a particular, see Figure 1). The images were presented in twelve (subjects TE and ZK) or eight (the remaining 8 subjects) 40-second-long epochs separated by 10-second rest intervals of a grey screen with fixation. During each epoch, the subject was presented only with images of faces and houses (no blanks, fixation screens, etc. were used), with the stimulus switching between face and house at irregular intervals —approximately every several seconds. The numbers of switches for each subject are summarized in Table 1.

Throughout the experiment, the subjects were requested to fixate at a black point in the center of the screen and report the stimulus category switches by pressing the button corresponding to the category that appeared (i.e., face or house) with the right hand. The MEG experiment used in our study served as a control condition in a larger emotional binocular rivalry experiment.

### 2.1. Data acquisition and preprocessing

MEG signals were recorded using 275-sensor whole-head CTF-275 system by VSM MedTech Ltd. Coquitlam, Canada. Because of a failure of one of the sensors, only 274 channels were recorded. All the sensors were 2nd-order axial gradiometers. The data was sampled at 600 Hz.

For computational efficiency reasons, the MEG signals were downsampled to 60 Hz. Then they were segmented into intervals of [ − 0.33 1] seconds or [ − 20 60] samples around the stimulus switch. Next, each interval was baseline corrected by subtracting the average of the first 20 samples from each sample in the interval. In this manner for each subject, we obtained several dozens of signals, each containing 274 (number of channels) ∗ 81 (number of time slices) values. Each of the signals was associated with class label “face” if it was recorded while stimulus switched from house to face and with class label “house” otherwise.

## 3. Fisher LDA-BASED FRAMEWORK FOR FUNCTIONAL BRAIN STATE INFERENCE

In a classical Fisher LDA setup, one is given two sets of scalars, *X* = {*x*_1_, *x*_2_, …,*x_n_*} and *Y* = {*y*_1_, *y*_2_, …,*y_m_*}, and the Fisher separation measure is given by

(1) $$d(x,y)=\frac{\left|\mu_{x}-\mu_{y}\right|}{\sqrt{\sigma_{x}^{2}+\sigma_{y}^{2}}},$$

where μ_x_ and μ_y_ are means and σ_x_ and σ_y_ are standard deviations of the two sets. The separation measure quantifies the “distinctiveness” of the two sets and can be thought of as signal-to-noise ratio of the associated classification problem.

For two sets of *K* -dimensional column vectors (representing labeled samples of two classes), X = {x_1_, x_2_, …,x_*n*_} and Y = {*y*_1_, *y*_2_, …,*y_n_*}, the direction p_f_ in the *k* -dimensional space that maximizes the Fisher separation between the projections of X and Y,

(2) $$p_{f}={arg}_{p}\,\;\max d(p^{t}X,p^{t}Y),$$

is given by

(3) $$p_{f}=S^{-1}(m_{x}-m_{y}),$$

where Σ = Σ_*x*_ + Σ_*y*_ is the sum of covariance matrices for X and Y and μ_*x*_, μ_y_—vector means of X and Y (see [24] for details). The inversion of Σ is problematic when the dimensionality of Σ is high and the number of observations is small. In that case, Σ is singular or close to singular, due to dimensions where the variance is zero or very small, and the inversion leads to large errors in the estimation of correct values even for dimensions where the variance is large.

Below, we extend this approach to temporal signals and address the singularity of the covariance matrix.

Following the MEG data preprocessing, we obtain a set of labeled signals, each signal being a matrix of 274 channels sampled at 81 consecutive time points (timeslices). Our main goal is to develop a method for inferring correct label from the signal matrix.

We assume a time-point-wise correspondence among the signals (the assumption is partially justified by the fact that the segmentation is timelocked to the stimulus). This assumption implies entrywise correspondence of the signal matrices, allowing us to treat each signal as a point in a 274 ∗ 81 -dimensional feature space. Thus, we can formulate our inference problem as a high-dimensional pattern classification problem.

Such high-dimensional classification problem poses 2 challenges:

1. feature selection —selecting a small subset of the 274 ∗ 81 -dimensional feature set that is most informative of the signal label.
2. classifier construction —building robust classifier from the selected feature subset.

### 3.1. Feature selection

There are many possible strategies for the feature selection step. In this study, we employed a very simple strategy of selecting the set of 274MEG sensor readings from a single most predictive time-point as a feature set for the classifier construction step (i.e., selecting the most predictive column from the 274 by 81 feature matrix). This reduces the dimension of the data from 274 ∗ 81 to 274. We evaluate the predictiveness of each timepoint by evaluating the performance of the resulting classifier using 100-fold cross-validation on all the data available.

### 3.2. Classifier construction

Once a set of 274 features is selected, one needs to construct a classifier for 274-dimensional vectors using a set of several dozens of labeled examples. We construct the classifier by computing from the labeled examples the optimal projection direction p_f_ in the 274-dimensional space using regularized Fisher LDA (see above). A new sample s is classified by projecting it onto p_f_ and applying a simple nearest-neighbor rule: for two classes X (faces) and Y (houses), decide that s belongs to X if

(4) $$\left|p_{f}^{t}s-p_{f}^{t}m_{x}|<|p_{f}^{t}s-p_{f}^{t}m_{y}\right|$$

and that s belongs to Y otherwise.

**Regularization technique**

We construct the classifier using Fisher LDA with slightly modified version of regularization described in [25]:

(5) $$\Sigma^{*}=S+\lambda e_{\max}I,$$

where *e*_max_ is the largest eigenvalue of the covariance matrix. Normalizing the second term of (5) by *e*_max_ allows a heuristic estimation of the relation between λ and the condition number of Σ. To illustrate this, let us assume that Σ is diagonal; in which case, its entries along the main diagonal are its eigenvalues. The condition number *c* of Σ_∗_ is then given by

(6) $$c=\frac{e_{\max}+\lambda e_{\max}}{e_{\min}+\lambda e_{\max}},$$

where *e*_min_ is the lowest eigenvalue of Σ. Since in our case the number of data samples is less than the data dimension, Σ is degenerate and has the lowest eigenvalue *e*_min_ = 0. Substituting zero for *e*_min_ in (6) gives us the relation between λ and the condition number

(7) $$c=\frac{1+\lambda }{\lambda }.$$

While (7) holds strictly only if Σ is diagonal, it can be used for heuristic approximation of *c* as a function of λ for any degenerate covariance matrix.

### 3.3. Relationships between λ and time

We argue that relations among λ, timepoint index *t*, and , 3, 5, 6, and 7. Please check. the classifier accuracy (estimated, e.g., by cross-validation) provide a wealth of information on both , 6, and 7. Please check. statistical and biological aspects of the problem (see the results section). This information can be utilized to guide feature selection, and evaluate data quality and other tasks. The current version of the proposed mental state inference technique uses this information to perform a very simple optimization —it selects the combination of *t* and λ yielding the lowest prediction error estimate.

The final classification of each signal is performed by doing single timepoint classification using the values of *t* and λ that minimize the estimated error.

### 3.4. Computational experiments

We estimated the classifier accuracy for each timeslice in the interval [ − 0.33 1] seconds and each value of the regularization parameter λ ∈[10^−5^ ,1]. According to (7), the lower limit of λ = 10^−5^ yields regularized matrix Σ^∗^ with condition number of order of magnitude 10^5^, which is the largest value for which the computation of the inverse of Σ + λe_max_I is still numerically stable. Using the values from the lower part of the range corresponds to the fixed diagonal regularization proposed in [26]. 300 values of λ were sampled uniformly on the logarithmic scale (i.e., the ratio of the two successive samples was constant) from the interval [ 10^−5^ 1].

For each timeslice and each value of λ, the classifier accuracy was estimated with 100-fold cross-validation using all the data available. In each iteration of the cross-validation, 80% of the data was used for training the classifier and 20% for testing.

## 4. RESULTS

### 4.1. Overall error rates

**Figure 2:**
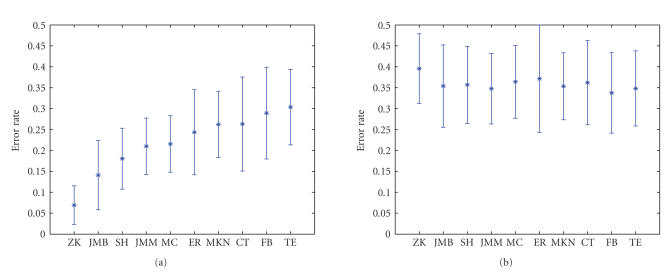
(a) Classifier error rates for all 10 subjects; regularization parameter and the input time slice were selected to minimize the classification error using 100-fold cross-validation. (b) Control results obtained using the same algorithm on data with randomly scrambled target labels; both plots show average error estimated using 100-fold cross-validation; error bars denote 1-std-wide margin around the estimate.

The lowest (over all timeslices and regularization parameter values) error rates achieved for each subject are summarized in Figure 2. Since minimizing the error over any free parameters biases, the error estimate downwards; we compare the estimated error to the estimate obtained by applying exactly the same algorithm to the data with randomly scrambled class labels (see Figure 2(b)). The difference between the mean error estimates is significant for all subjects (*P*<10^−3^ for all subjects, estimated using Student's t-test).

### 4.2. Relation between classifier error and regularization parameter

**Figure 3:**
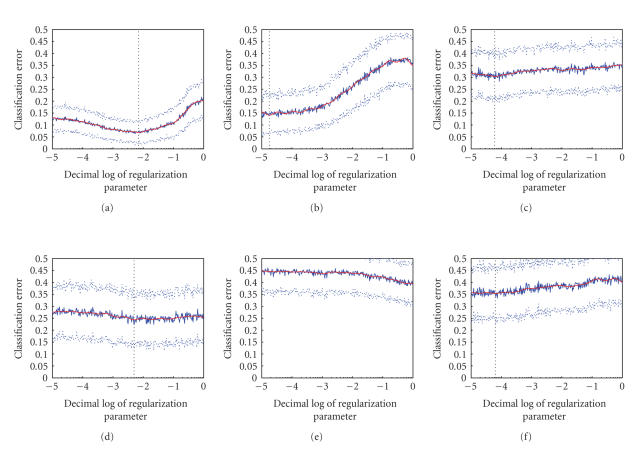
Prediction error at the best time slice versus log of regularization parameter. (a), (b) predictable subjects —ZK and JMB. (c), (d) unpredictable subjects —TE and ER. (e), (f) control experiments, in which category labels for subjects ZK and JMB were randomly scrambled before constructing the classifier. Classifier's prediction error was estimated using 100-fold cross-validation on 20% of the data. Dotted lines denote 1-std-wide margins of the estimate. The dotted vertical line marks the global minimum of the smoothed error estimate (smooth red line).

For a classification problem that uses regularization, one typically expects that the (estimated) classifier error as a function of regularization parameter exhibits a clear global minimum. In our case, the classification error when plotted against the regularization parameter clearly revealed such minimum in some subjects, while in others it remained completely flat (see Figure 3). Subjects that produced such flat plots also tended to achieve lower classification accuracy, which lead us to speculate the convexity of the plot might be indicative of the amount of noise in the data. One might think of the phenomenon in terms of a continuum of different signal-to-noise ratios: the more noise there is in the subject's data, the more similar it is to the random controls, both in terms of minimal achievable error and in terms of convexity of the plot.

### 4.3. Best separating weight maps

The set weights assigned to the MEG channels by the regularized Fisher LDA analysis can be interpreted as a weight map over the MEG helmet surface indicating the contribution of each point to the classification decision.

**Figure 4:**
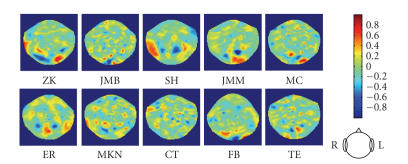
MEG sensor weight maps for the 10 subjects. Each map corresponds to the time slice and the regularization value that yield lowest prediction error estimate for the given subject. The maps are presented in the order of increasing classifier error (from left to right and from top to bottom).

**Figure 5:**
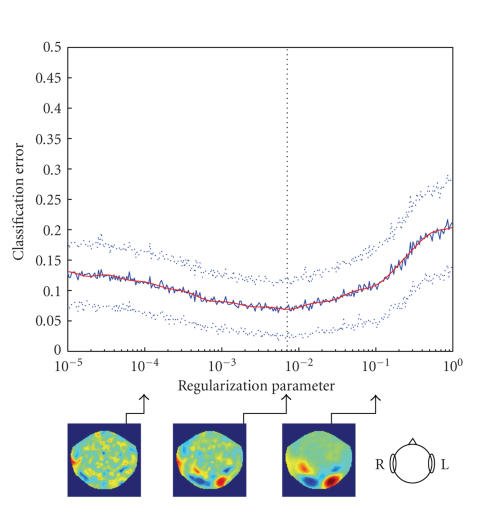
Error rate as a function of regularization parameter for subject ZK. Solid blue line denotes the average error rate over 100-fold cross-validation, dotted lines mark 1-std-wide margin; the vertical line marks the minimum of the smoothed error rate (red line). Three plots below show the distribution of sensor weights corresponding to different values of the regularization parameter.

We examined the weight maps obtained for the combination of λ and timeslice that yield the lowest estimated prediction error. The maps display a prominent structure consisting of several small clusters of interleaved positive and negative weights (see Figure 4). As expected from animal single unit and fMRI human studies [27], this structure is fairly localized to occipitotemporal regions that might correspond to a neural source in the fusiform gyrus. The structure seems to be more clearly exhibited in the predictable subjects. We also investigated the relation between the value of λ and the structure of corresponding weight maps. As one could have expected, increasing the regularization parameter causes the resulting optimal weight maps to become smoother (see Figure 5).

### 4.4. Spatiotemporal structure of the signal and its relation to the regularization parameter

**Figure 6:**
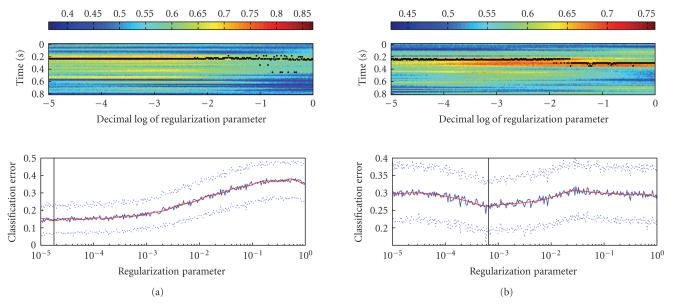
(a) Temporal stability of the best separating timeslice as a function of regularization parameter for subject JMB. The upper plot shows the accuracy of the classifier as a function of timeslice and regularization parameter. The accuracy is denoted by the color according to the colorbar above the plot. Timeslice yielding maximum accuracy for each value of the regularization parameter is marked by a black dot. The lower part of the plot shows the best (over all timeslices) error plotted against the regularization parameter using the same timescale as the upper part. (b) Same as (a) but for subject MKN.

Another item of particular interest is the temporal structure of the signal and its relation to the regularization parameter. We discovered that the stability of the best separating timeslice as a function of regularization and classifier performance as a function of regularization are closely related. The temporal location of the best separating timeslice tends to be more stable for the λ values that yield lower classification error (see Figure 6).

The figure also reveals that the most informative timeslices are located approximately 0.2 seconds after the stimulus switch. This finding is consistent with previous findings about the N170 wave —an increase in negative potential at the parietal parts of the scalp, approximately 0.17 seconds after stimulus presentation [28, 29]. One can also see that there are other timeslices in addition to those located at 0.2 seconds, that can potentially .'' Please check. contribute to improved classification (e.g., the timeslices located near 0.32 and 0.5 seconds in Figure 6(b)).

### 4.5. Comparison to other classification techniques

**Figure 7:**
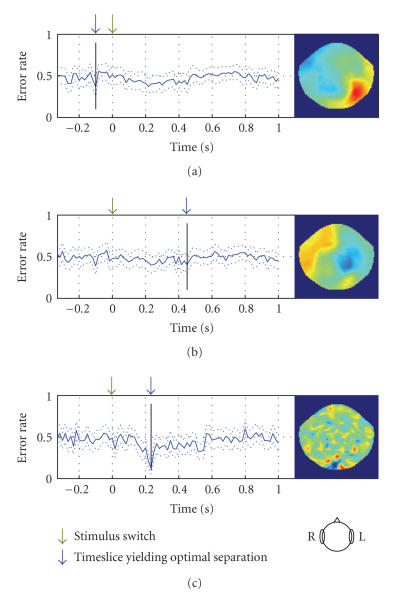
Comparison between different linear discrimination methods for subject JMB. (a) Using sensorwise difference of mean signals for two conditions as weights. (b) Same as (a) but the weight of each sensor is normalized by the variance of the signal at that sensor. (c) Regularized Fisher linear discriminant analysis. The plots depict error estimate of the classifier as a function of time slice of MEG signal to which it was applied. Dotted lines denote 1-std-wide margin around the estimate. The maps depict distribution of weights over the scalp (flattened helmet viewed from above) at the time slice that yields best separation (marked by blue arrow).

Finally, we compared regularized Fisher LDA to two other more . Please check. straightforward techniques: sensorwise difference of average signals for faces and houses and sensorwise difference normalized by sensorwise signal variance (see Figure 7). Note that each classifier attains best separation at a different time. Regularized Fisher linear discriminant differs from the other methods in 3 aspects: (1) it achieves much lower error rate: 14% against 37% and 39% for the other methods; (2) the global minimum of the error function is much more clearly localized in time; (3) the corresponding weights map shows a prominent pattern localized to the sensors located over occipital region of the brain.

### 4.6. Neuronal basis of the classification

The differential neuronal activity that allows distinguishing between the two types of stimulus switches can be attributed to the differences in visual processing of the stimulus, the differences in the planning and execution of the response motor task, or both. However, observations support the notion that differences in activity detected by the classifier are predominantly of the visual category processing nature. First, the classifier accuracy when plotted as a function of time peaks at about 200 milliseconds which is consistent with other findings regarding the N170 wave and its role in face processing [28, 29]. As expected from N170 distribution, weight maps resulting from the presented classification tend to assign higher importance to sensors located over the occipital and temporal lobes. Finally, behaviorally there was no significant difference between average reaction times for the two stimulus categories suggesting that for both stimulus classes the motor-related neuronal activity is similar.

## 5. CONCLUSIONS

We have proposed a new framework for the functional brain state inference problem. The framework utilizes temporal information present in EEG and MEG signals and is particularly adapted to the needs of functional neuroimaging. Application of the framework to MEG data suggests that the relation between regularization parameter and temporal profile of the classifier reveals a lot of structure that can be utilized for improving classification accuracy. This structure can be exploited to construct more accurate classifiers, for example, by fusing information across different combinations of regularization parameters and times. The proposed classification framework opens a new horizon for whole-brain functional imaging where combined temporal and spatial characteristics of brain signals can reveal the underlying physiological mechanism of an individual's functional state. It can further promote studies on internally driven mental events such as spontaneous switching in awareness, emerging of volition, and formulation of intention.

## Figures and Tables

**Table 1:** Number of training samples for each subject

Subject	CT	ER	FB	JMB	JMM	MC	MKN	SH	TE	ZK
No. of switches from house to face	42	39	47	48	74	65	80	55	57	72
No. of switches from face to house	39	36	46	44	68	61	76	56	53	66
